# Multi-perspective analysis of skin microbiota in elderly eczema patients

**DOI:** 10.1371/journal.pone.0318240

**Published:** 2025-03-27

**Authors:** Dake Dong, Qianjie Wu, Zijun Wang, Pengfei Xu, Tianqing Zhu, Ting Yang, Zhenghua Gu, Liang Zhang, Zitao Guo

**Affiliations:** 1 Department of Dermatology, Affiliated Hospital of Jiangnan University, Wuxi, China; 2 Wuxi Medical College, Jiangnan University, Wuxi, China; 3 School of Biotechnology, Jiangnan University, Wuxi, China; 4 National Engineering Research Center of Cereal Fermentation and Food Biomanufacturing, Jiangnan University, Wuxi, China; 5 Wuxi Food Safety Inspection and Test Center, Technology Innovation Center of Special Food for State Market Regulation, Wuxi, China; 6 School of Food and Biological Engineering, Jiangsu University, Zhenjiang, China; Saveetha Medical College and Hospital, INDIA

## Abstract

Eczema is a common inflammatory skin disease in elderly people. It not only causes physical damage to elderly people but also seriously affects their mental health. The skin microbiota plays a vital role in the development of skin disease. However, relatively few studies have investigated the characteristics of the skin microbiota in elderly eczema patients. In this study, the differences in the composition of the skin microbiota between lesion sites and healthy sites, between exposed sites and unexposed sites, and between elderly and younger eczema sites were analyzed, aiming to characterize the skin microbiota in elderly eczema patients from multiple perspectives and provide a basis for clinical diagnosis and treatment. The results indicated that the species richness of elderly eczema patients was greater than that of younger eczema patients. There was no significant difference between groups at the phylum level. At the genus level, the abundance of *Staphylococcus* significantly increased in the lesion sites of the elderly group. Compared with the younger eczema group, the elderly eczema group had greater abundances of *Paracoccus*, *Deinococcus_B*, *Kaistella*, *Escherichia*_710834, and *Chryseobacterium*_796703. These findings indicated that more attention should be given to the roles of *Kaistella* and *Streptococcus* in elderly eczema patients because *Kaistella* was the only genus among the 20 most abundant genera that was closely related to the EASI scores. Moreover, correlation analysis suggested that many genera had a positive relationship with *Streptococcus*. The results provide basic microbiological data for physicians treating elderly patients with eczema.

## Introduction

Aging is currently a major trend in society development and an unprecedented challenge in the history of human development [1]. According to the World Population Prospects (2015 Revision), the global population aged 60 years and over is projected to increase by 56%, from 901 million to 1.4 billion, between 2015 and 2030. By 2050, the global older population is expected to reach 2.1 billion [2]. The Asia-Pacific region is the fastest-aged region in the world, with a quarter of the population predicted to be over the age of 60 by 2050. According to the National Bureau of Statistics of China, in 2020, the total number of elderly people aged 60 and above in the mainland region was 264 million, accounting for 18.7% of the total population. It is expected that by 2050, there will be approximately 500 million people (https://www.stats.gov.cn/sj/, access date: 2024/09/18). The skin is the largest organ of the body and is the first line of defense between the internal and external environment of our body [3]. Aging affects all organs, including the skin, which is the most affected. With increasing global aging, the incidence of skin diseases in elderly individuals is also increasing annually [4]. Eczema, a chronic inflammatory skin condition in which the skin becomes red, inflamed, itchy and scaly, can develop easily in older adults. The main clinical feature of eczema is itching. The rash pattern is diverse and can include erythema, papules, blisters, oozing, or crusting. In the chronic stage, the skin is dry and desquamated, keratinized, and mossy. Histopathology shows spongiotic edema and different degrees of spinous layer hypertrophy [5]. In the long-term absence of effective treatment, elderly people may suffer from itching. It is easy for elderly people to develop negative emotions such as nervousness, anxiety, and depression, which seriously affect their quality of life.

Barrier function is one of the main functions of the skin [6]. A healthy skin microbiota may provide multiple protections for the skin barrier, which consists of bacteria, fungi, and viruses that colonize the surface of the epidermis. First, the skin microbiota could provide a bacterial barrier for the skin. Skin bacteria can be mainly divided into resident bacteria and transient bacteria. Resident bacteria can inhibit the overpopulation of transient bacteria and the invasion of pathogenic bacteria through various mechanisms, such as nutrient competition, secretion of antimicrobial peptides, and interference with the community sensing system. For example, specific *Staphylococcus humanis* strains can produce thiopeptide antibiotics that inhibit the activity of *Staphylococcus aureus* [7]. Next, the skin microbiota plays an active role in maintaining the physical barrier of the skin. It has been found that the skin microbiota can promote keratinocyte formation by interfering with keratinocyte differentiation signaling pathways. Some *Staphylococcus epidermidis* can secrete sphingomyelinase to convert lamellar lipids on the surface of the skin into ceramides to maintain the normal function of the stratum corneum [8]. Moreover, the skin microbiota is involved in the regulation of the skin immune barrier. On the one hand, certain metabolites of the skin microbiota can directly inhibit skin cell inflammation. On the other hand, these metabolites could indirectly strengthen the cutaneous immune barrier by stimulating skin cells to produce defensins, etc. Recently, a study showed that propionic acid, a lipid metabolite of *Cutibacterium acnes* (*C. acnes*), could inhibit the production of interleukin-33 (IL-33) by keratinocytes. The topical external application of propionic acid could reduce skin inflammation and itching scores in patients with atopic dermatitis [9]. In summary, the skin microbiota plays a vital role in maintaining the skin barrier.

Disturbance of the skin microbiota has been implicated in the development of various kinds of skin diseases, including acne, atopic dermatitis, and psoriasis [10]. The most common skin conditions in older people are eczema, skin infections and itching [11]. However, there is currently a lack of studies exploring the role of the skin microbiota in elderly individuals with eczema. This may be mainly because elderly eczema was not given enough attention when society was less aging, resulting in less basic data on the characteristics of their skin microbiota. In this study, to provide more insights into the role of the skin microbiota in elderly eczema for related researchers, the characteristics of elderly eczema patients were investigated by 16S rRNA gene sequencing. The differences in the composition of the skin microbiota between lesion sites and healthy sites, between exposed sites and unexposed sites, and between elderly and younger eczema sites were analyzed. The results of this study would provide basic microbiological data for physicians treating elderly patients with eczema.

## Materials and methods

### Subjects

The subjects were recruited from the eczema patients who visited the Department of Dermatology at the Affiliated Hospital of Jiangnan University between June 15 and October 15, 2023. Patients who had a history of any topical or oral therapeutic medication in the past two weeks, psychiatric disorders, pregnancy or breastfeeding, cardiovascular or cerebrovascular disorders, severe skin infections, severe diabetes mellitus, or combined malignancies were excluded. Those over the age of 60 were included in the older group, and the rest were included in the younger group. In addition, the sampling sites were divided into exposed areas (including the face and limbs) and unexposed areas (including the chest, back and buttocks). The study was approved by the Ethics Committee of Jiangnan University Affiliated Hospital (Ethics Approval No. LS2023058), and consented by the subjects, who all signed informed consent.

### Clinical Eczema area and severity index (EASI) scores and sample collection

The EASI score was used to evaluate the illness condition of the patients as previously described [12]. A sterile saline-soaked swab was used to collect skin samples. The sampler wore masks and gloves for the entire duration of the sampling process, which was carried out in a room with a constant temperature and humidity (25°C, 40-50%). During sampling, the sampler rapidly rubbed approximately 40 times in a 4 cm radius around the sampling site. Samples were taken from healthy skin on the symmetrical side of the lesion site as a control group. The number of samples taken from each patient’s skin surface varies. If the patient has eczema in only one location, a single sample was taken from the lesion site, and one sample was also collected from the healthy skin on the symmetrical side of the patient. If the patient has eczema in different areas, we sampled based on the body regions (primarily categorized into head, arms, chest and back, buttocks, and legs), and the healthy skin on the symmetrical side of the lesion site was also sampled. Additionally, to avoid taking too many samples from one patient, the number of samples collected from both the affected and healthy areas was limited to a maximum of two, respectively. All samples used were collected by the same person to avoid manipulating the samples. The swabs were then placed in a sterile tube and transferred to a -80°C freezer within 30 min.

### DNA extraction and sequencing

DNA from the samples was extracted using a universal DNA extraction kit (HiPure Blood & Tissue DNA Kit) and quantified using a Thermo Scientific NanoDrop 2000C spectrophotometer. After that, the DNA integrity was detected by agarose gel electrophoresis. The primers for the V3-V4 region, 341F (5’-CCTAYGGGRBGCASCAG-3’) and 806R (5’-GGACTACNNGGGTATCTAAT-3’), were used for amplification. Then, the PCR products were detected by gel electrophoresis, and the target fragments were purified and recovered. Library construction and quality control, poor-quality removal and splicing contamination, sequence ligation and assembly, and sequencing were carried out as described in previous literature [13].

### Bioinformatic analysis

The raw data were filtered to generate high-quality clean reads as previously described [14]. The denoising method of DADA2 (divisive amplicon denoising algorithm) in the software QIIME2 was used to obtain amplicon sequence variants (ASVs), and the sequences of the ASVs were 100% similar. OTU representative sequences were aligned against the database (Greengene V201305) for taxonomic annotation by RDP classifier (v2.2) software (sequence identity was set to 0.6). The Chao and Shannon indices were selected to characterize the alpha diversity of the skin microbiota of the samples, while the beta diversity analysis was performed with QIIME software (v1.80) and displayed by principal coordinate analysis (PCoA) based on the Bray‒Curtis distance [15]. The composition of the skin microbiota of the samples was analyzed at the phylum and genus levels. The significant differences at different levels between groups were evaluated by the relative abundance of bacteria. In addition, the relationship between the skin microbiota and the EASI score was assessed by Spearman correlation analysis, and the results are displayed as a heatmap. Moreover, a correlation analysis between bacteria was also conducted. Finally, PICRUSt2 (Phylogenetic Investigation of Communities by Reconstruction of Unobserved States, v2.3.0-b) was used to predict the functional abundance of the skin microbial community based on the Kyoto Encyclopedia of Genes and Genomes (KEGG) database [16].

### Statistical analysis

The data are expressed as the means ±  standard deviations (SD). One-way analysis of variance (ANOVA) was used to determine the significant differences between groups by using SPSS 25.0 software (SPSS Inc., Chicago, IL, United States) according to previous study [17]. The confidence interval was 95%, and a value of *p* <  0.05 was considered to indicate statistical significance.

## Results

### Cohort characterization

Ultimately, 64 patients were enrolled in the trial. Among them, 24 subjects in the older group and 40 in the younger group were included. A total of 127 samples were sequenced after DNA quality testing. The number of lesion and healthy samples in the older group was 31 and 28, respectively, while the number in the younger group was the same (n = 34). Clinical data suggested that most cases of eczema in patients are in exposed areas (Table 1). In the older group, the number of samples in the exposed and unexposed areas of lesion sites was 23 and 8, respectively. In the younger group, a total of 23 and 11 samples from the exposed and unexposed lesion sites, respectively, were sequenced.

**Table 1.** Characteristics of the cohort.

Group	Number of subjects		Exposed area	Unexposed area
Older group ( ≥ 60 years of age)	24	Lesion sites	23	8
		Healthy sites	19	9
Younger group ( < 60 years of age)	40	Lesion sites	23	11
		Healthy sites	22	12

### The diversity of the skin microbiota

**Fig 1:**
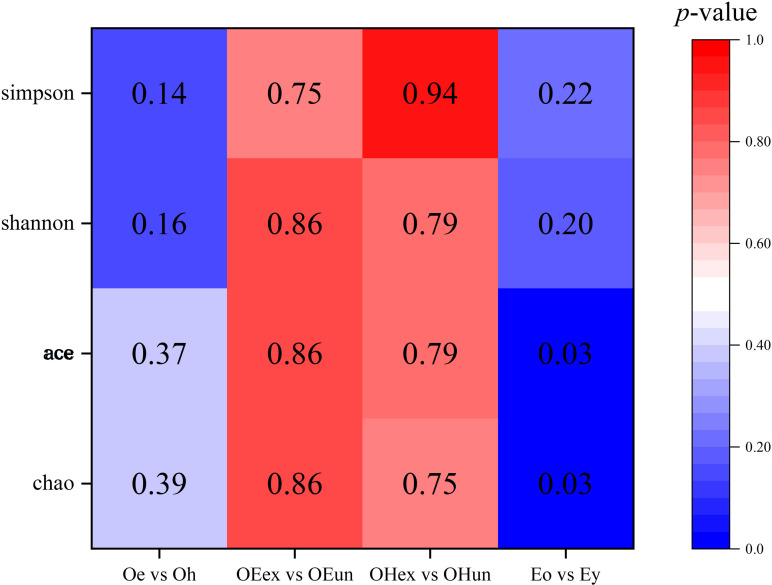
Statistical analysis of the p values of the differences in alpha diversity between groups. Oe, elderly eczema sites; Oh, elderly health sites; OEex, elderly eczema exposed sites; OEun, elderly eczema unexposed sites; OHex, elderly healthy exposed sites; OHun, elderly healthy unexposed sites; Eo, older group eczema sites; Ey, younger group eczema sites.

**Fig 2:**
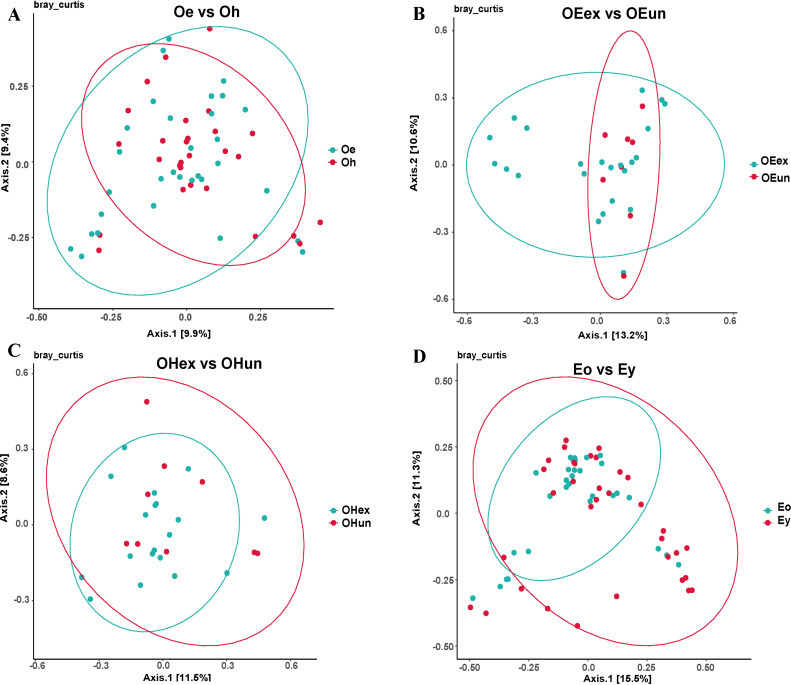
The beta diversity of the skin microbiota between groups. Oe, elderly eczema sites; Oh, elderly health sites; OEex, elderly eczema exposed sites; OEun, elderly eczema unexposed sites; OHex, elderly healthy exposed sites; OHun, elderly healthy unexposed sites; Eo, older group eczema sites; Ey, younger group eczema sites.

The alpha diversity of the skin microbiota was represented by a heatmap based on the Chao, ACE, Shannon and Simpson indices (Fig 1). The number shown in the figure is the *p* value after statistical analysis. There were no significant differences in alpha diversity between groups in the older group (Fig 1, Table 2). The *p* values of lesion and healthy sites were all above 0.1, while those of exposed and unexposed sites exceeded 0.7. For the lesion sites between the older and younger groups, the significant differences (*p* = 0.03) in the ACE and Chao indices suggested that there were significant differences in the species richness between the two age groups. The beta diversity is shown in Fig 2. Although the intergroups did not form two completely separated parts, there were differences between some samples. In particular, there were differences between the exposed and unexposed sites of the older eczema group and between the older eczema group and the younger eczema group (Fig 2 B and D).

**Table 2.** The alpha diversity of the skin microbiota in each group.

Groups	ace	chao	shannon	simpson
Oe	777.30 ± 327.16	765.39 ± 320.57	4.63 ± 1.38	0.83 ± 0.13
Oh	913.74 ± 491.34	897.59 ± 480.80	5.18 ± 1.85	0.85 ± 0.19
OEex	773.45 ± 294.85	760.79 ± 288.56	4.64 ± 1.33	0.83 ± 0.12
OEun	788.39 ± 405.78	778.58 ± 398.24	4.60 ± 1.52	0.81 ± 0.15
OHex	920.27 ± 464.92	902.88 ± 453.45	5.26 ± 1.75	0.86 ± 0.18
OHun	899.97 ± 542.66	886.41 ± 533.78	5.02 ± 2.03	0.82 ± 0.20
Eo	777.3 ± 327.16	765.39 ± 320.57	4.63 ± 1.38	0.83 ± 0.13
Ey	571.42 ± 291.00	566.17 ± 286.50	3.91 ± 1.70	0.73 ± 0.24

Oe, elderly eczema sites; Oh, elderly health sites; OEex, elderly eczema exposed sites; OEun, elderly eczema unexposed sites; OHex, elderly healthy exposed sites; OHun, elderly healthy unexposed sites; Eo, older group eczema sites; Ey, younger group eczema sites.

### The composition of the skin microbiota

**Fig 3:**
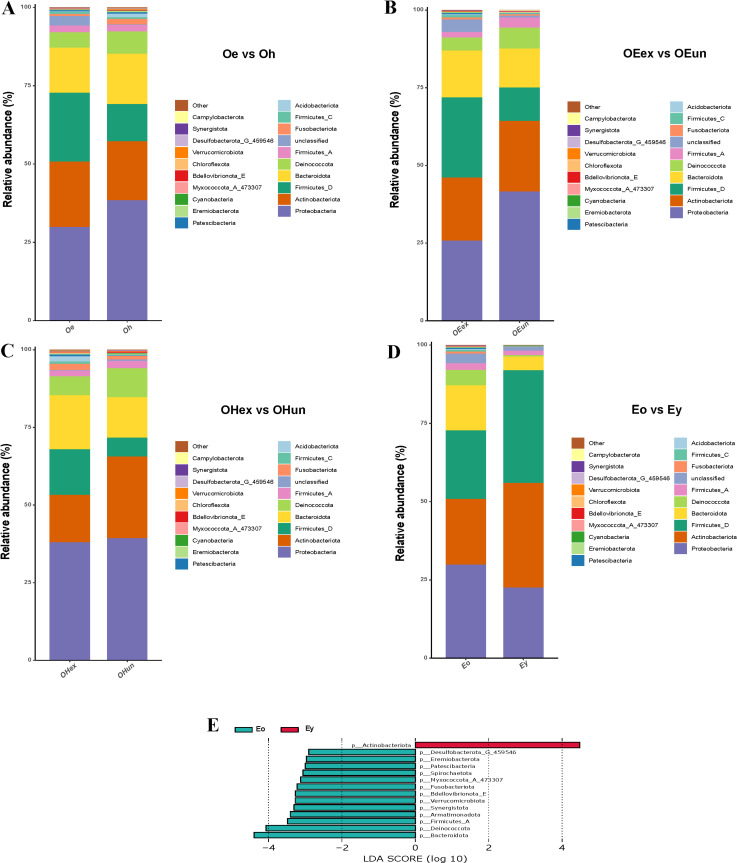
Comparison of the skin microbiota composition at the phylum level between groups. A, Oe vs Oh; B, OEex vs OEun; C, OHex vs OHun; D and E: Eo vs Ey. Oe, elderly eczema sites; Oh, elderly health sites; OEex, elderly eczema exposed sites; OEun, elderly eczema unexposed sites; OHex, elderly healthy exposed sites; OHun, elderly healthy unexposed sites; Eo, older group eczema sites; Ey, younger group eczema sites.

**Fig 4:**
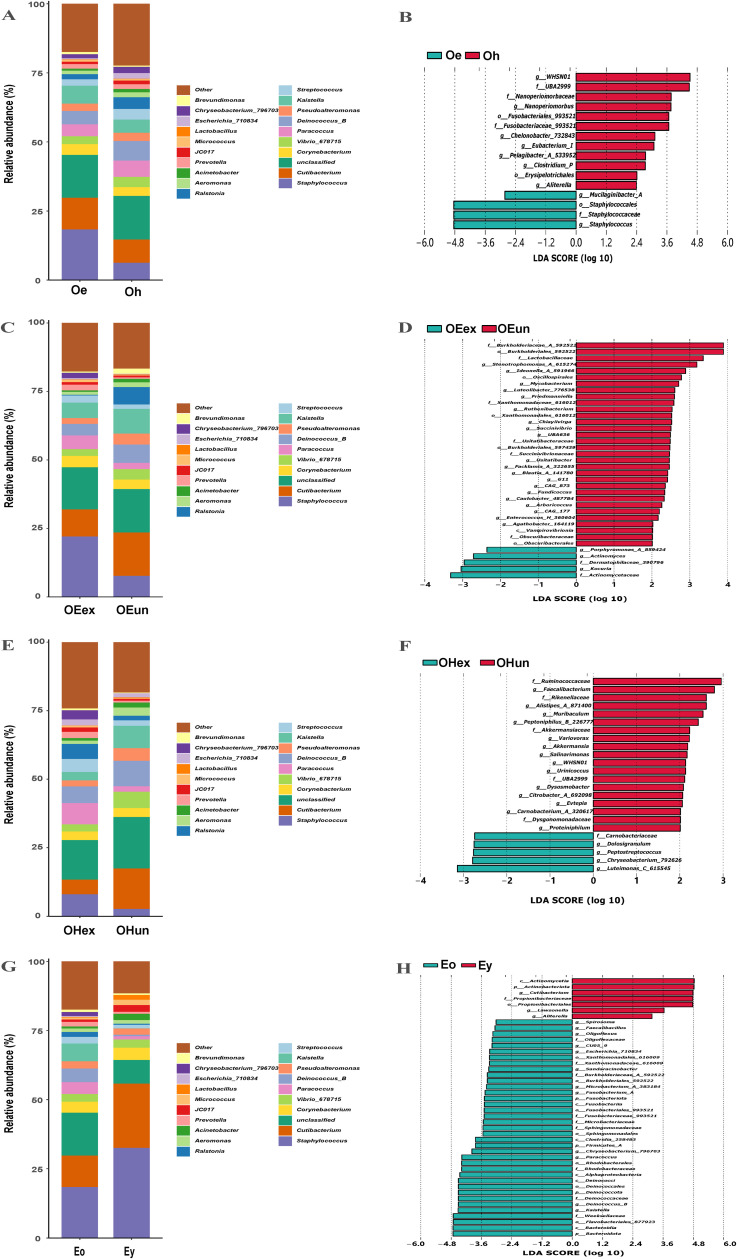
Comparison of the skin microbiota composition at the genus level between groups. A and B: Oe vs Oh; C and D: OEex vs OEun; E and F: OHex vs OHun; G and H: Eo vs Ey. Oe, elderly eczema sites; Oh, elderly health sites; OEex, elderly eczema exposed sites; OEun, elderly eczema unexposed sites; OHex, elderly healthy exposed sites; OHun, elderly healthy unexposed sites; Eo, older group eczema sites; Ey, younger group eczema sites.

At the phylum level, the skin microbiota was mainly composed of Proteobacteria, Actinobacteriota, Firmicutes_D, Bacteroidota, and Deinococcota (Fig 3). The sum relative abundance of these five genera was approximately 80%. In the older groups, there were differences in the composition of the different groups (Fig 3 A–C). However, according to the results of the statistical analysis of the data, there was only a trend of change. The differences between the groups were not significant. Among the top 10 phyla in terms of relative abundance, there were significant differences in the relative abundance of five phyla between the eczema older and eczema younger groups. They included Actinobacteriota, Bacteroidota, Deinococcota, Firmicutes_A, and Fusobacteriota. The relative abundance of Actinobacteriota in the younger eczema group was greater than that in the older eczema group, while the relative abundance of the other four phyla decreased (Fig 3 D,E). At the genus level, the top 10 genera were *Staphylococcus*, *Cutibacterium*, *Corynebacterium*, *Vibrio_678715*, *Paracoccus*, *Deinococcus_B*, *Pseudoalteromonas*, *Kaistella*, *Streptococcus*, and *Ralstonia* (Fig 4). The relative abundances of 9 genera were significantly different between the lesion and healthy sites in the older group (Fig 4 A,B). The relative abundance of *Staphylococcus* sharply increased at lesion sites compared with healthy sites. Except for *Staphylococcus*, the other 8 genera were not the major genera. The relative abundances of these genera were outside the top 20 genera in the skin microbiota. In the lesion sites of older patients, there were significant differences in the relative abundance of 23 genera between the exposed sites and the unexposed sites. However, the relative abundances of these genera were not among the top 20 genera (Fig 4 C,D). Although there was no difference between the exposed sites and unexposed sites in terms of the relative abundance of *Staphylococcus*, the increasing trend of *Staphylococcus* abundance in the exposed sites deserves attention. Similar to the lesion site results, the 18 genera with significant differences between the exposed and unexposed sites among the healthy sites were all among the top 20 most abundant genera (Fig 4 E,F). For the older eczema group and the younger eczema group, there were significant differences in the relative abundance of many genera. Thus, the genera with LDA scores (log 10) above 3.0 are shown (Fig 4 H). Finally, a total of 15 genera with significant differences in relative abundance were found. Among them, 6 genera were included in the top 20 genera (Fig 4 G). These genera included *Cutibacterium*, *Escherichia_710834*, *Chryseobacterium_796703*, *Paracoccus*, *Deinococcus_B*, and *Kaistella*. The relative abundance of *Cutibacterium* in the younger eczema group was significantly greater than that in the older eczema group; however, the abundances of the other 4 genera were lower.

### Correlation analysis

**Fig 5:**
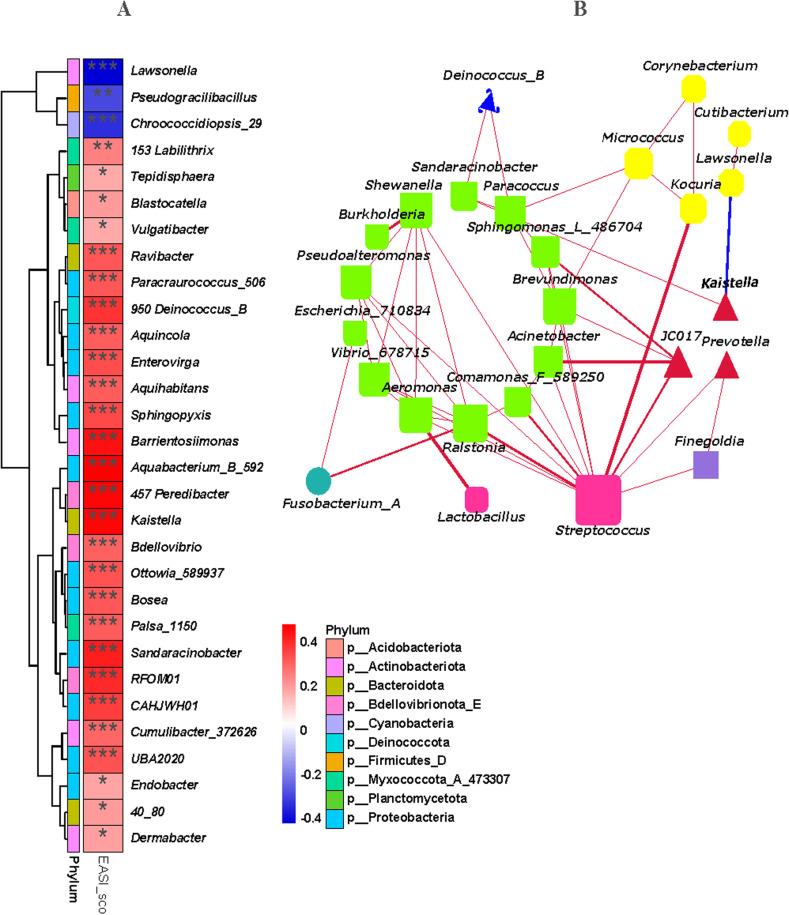
Spearman correlation analysis. A, The correlation between the skin microbiota at the genus level and the EASI score. B, The correlation between bacteria at the genus level.

At the genus level, correlation analyses were performed between bacteria and EASI scores as well as between bacteria and bacteria (Fig 5 A,B). These results indicated that 30 genera had a close relationship with the EASI score, and most of these genera had a positive relationship, while only 3 genera, namely, *Lawsonella*, *Pseudogracilibacillus*, and *Chroococcidiopsis_29,* exhibited a negative relationship. In addition, most of these genera belong to the phyla Proteobacteria (11 genera) and Actinobacteriota (6 genera). The number of genera in the other phyla was less than three. Among the 30 genera, however, only *Kaistella* was among the top 20 genera in the skin microbiota (Figs 4 and 5 A). The network between genera included 26 genera, which mainly belonged to the phyla Proteobacteria (green bottom-round-rectangle, which included 13 genera), Actinobacteriota (yellow cut-rectangle, which included 5 genera), Bacteroidota (red triangle, which included 3 genera), Firmicutes_D (pink round-rectangle, 2 genera), Fusobacteriota (blue‒green circle, 1 genus), Deinococcota (blue round-triangle, 1 genus), and Firmicutes_A (purple rectangle, 1 genera). The relationships between genera indicated that they were mostly positively correlated, and only a negative correlation was found between *Kaistella* and *Lawsonella*. Surprisingly, the genus *Streptococcus* had a close relationship with most of these genera (Fig 5 B).

### Predicted function of the skin microbiota

**Fig 6:**
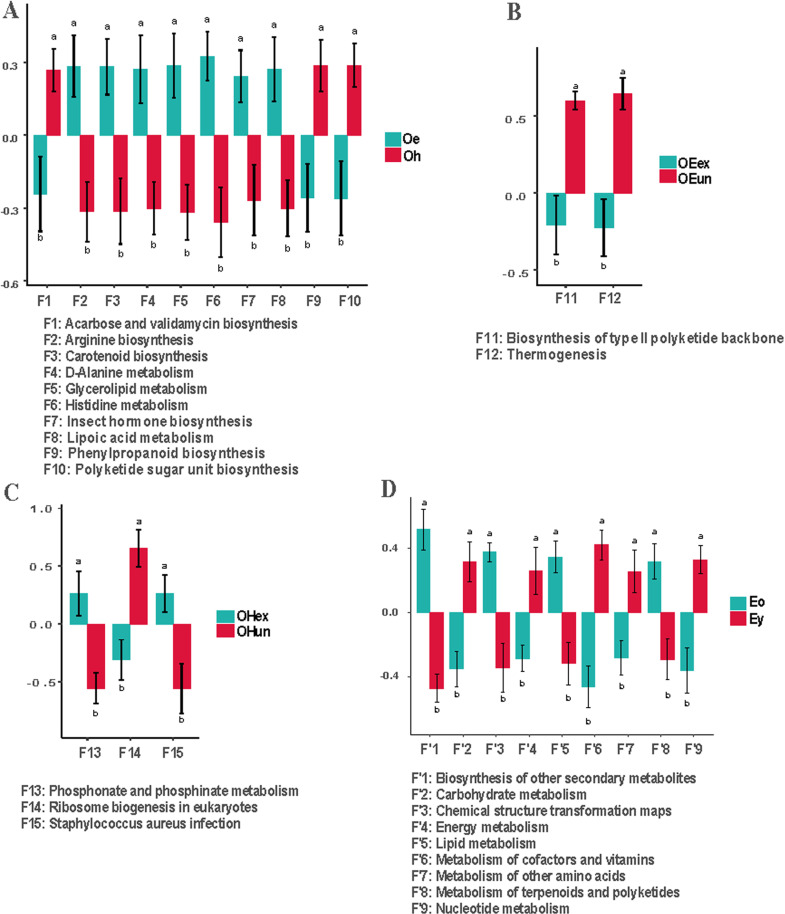
Comparison of the predicted functions of the skin microbiota between groups. A, Oe vs Oh; B, OEex vs OEun; C, Eo vs Ey, D, OHex vs OHun. Oe, elderly eczema sites; Oh, elderly health sites; OEex, elderly eczema exposed sites; OEun, elderly eczema unexposed sites; OHex, elderly healthy exposed sites; OHun, elderly healthy unexposed sites; Eo, older group eczema sites; Ey, younger group eczema sites.

The PICRUSt analyzes 16S rRNA gene sequencing data to infer the functional profiles of microbial communities. PICRUSt maps identified taxa to known functional genes using the extensive KEGG database. This approach enables us to estimate the abundance of different metabolic pathways, thereby providing valuable insights into the potential biological roles that the skin microbiome may play in skin health and disease. The detailed analysis steps were conducted according to the previous study [18]. Compared with the healthy sites in the older group, the lesion sites had a significant difference in level 3 of the KEGG pathway. A total of 10 significantly different predicted functions were found, among which the abundance of 7 predicted functions increased in the lesion site group. These pathways included arginine biosynthesis, carotenoid biosynthesis, D-alanine metabolism, glycerolipid metabolism, histidine metabolism, insect hormone biosynthesis, and lipoic acid metabolism (Fig 6 A). There were fewer significant differences between exposed sites and unexposed sites. In the older group, only 2 and 3 predicted functions were found to have significant differences between the exposed and unexposed sites in the eczema and healthy sites, respectively (Fig 6 B,C). The lack of significant differences in the diversity and composition of the skin microbiota between the exposed and unexposed sites might be responsible for these results (Figs 1-4). The differences between the older eczema group and the younger eczema group were statistically analyzed in level 2 of the KEGG pathway because too many predicted functions were significantly different in level 3 of the KEGG pathway. These results indicated that the two groups had a significant difference in the abundance of 9 predicted functions. The abundances of carbohydrate metabolism, energy metabolism, cofactor and vitamin metabolism, other amino acid metabolism, and nucleotide metabolism were greater in the younger eczema group than in the older eczema group (Fig 6 D).

## Discussion

Skin microbes are closely related to the structure of the skin, and each age group has its own characteristics. Fetal skin is sterile, and microbial colonization occurs during delivery, which may result in significant differences in skin microbiota composition depending on the mode of delivery [19]. With the gradual improvement in skin function at the beginning of life, the skin microbiome gradually develops and stabilizes within the first year of life [20]. In the adolescent stage, due to endocrine changes, skin oil secretion is vigorous, and the abundance of *C. acnes* begins to gradually increase, resulting in changes in the structure of the microbiota. Unless the external environment changes greatly, the skin microbiota of adults is relatively stable [21]. With increasing age, a variety of endogenous or exogenous influencing factors significantly influence skin structure and function, and these changes significantly impact the diversity and structure of the skin microbiota in elderly individuals [22]. The acceleration of global aging has led to an increase in the incidence of geriatric skin diseases. Eczema is a common inflammatory skin disease in elderly people who not only causes physical damage to elderly people but also seriously affects their mental health. The skin microbiota plays a vital role in the development of skin disease. However, relatively few studies have investigated the characteristics of the skin microbiota in elderly patients with eczema. In this study, the composition of the skin microbiota in elderly eczema patients was characterized by 16S rRNA gene sequencing, aiming to characterize the skin microbiota in elderly patients with eczema from multiple perspectives and provide a basis for clinical diagnosis and treatment. Finally, our results indicated that the roles of several key genera, such as *Staphylococcus*, *Kaistella,* and *Streptococcus*, in eczema in elderly individuals should receive increased attention.

The genus *Staphylococcus* is one of the most common commensal bacteria on the surface of human skin. A variety of staphylococci play important roles in the development and progression of skin diseases, the most studied of which are *Staphylococcus aureus* (*S. aureus*) and coagulase-negative staphylococci (CoNSs). *S. aureus* is a common human veterinary pathogen that causes a wide range of human infections and diseases, including skin infections and pneumonia [23,24]. Bacterial infection is reportedly an important cause of eczema dermatitis, especially *S. aureus* infection, which promotes the occurrence and development of this disease [25,26]. These results are consistent with our findings, as the increased abundance of *Staphylococcus* was a representative characteristic of elderly eczema patients (Fig 4 A-F). *S. aureus* can produce antigens composed of bacterial exotoxins or reverse transcription proteins, also known as superantigens (SAgs) [27]. SAgs can cause abnormal and excessive T-cell responses in mammalian hosts. Although they can be produced by a wide range of infectious microorganisms, SAgs produced by *S. aureus* are of particular importance in human disease. It can activate T lymphocytes in large numbers by binding to the Vβ chain of the T-cell receptor (TCR), release inflammatory transmitters, and cause superantigen-specific IgE synthesis. Finally, these effects could induce the formation of eczema-like changes in the skin or aggravate a preexisting rash. CoNSs are among the commensal bacteria of the skin, oral cavity, and intestinal tract [28]. They are regarded as nonpathogenic because most of them are able to establish a symbiotic relationship with the human body [29]. Moreover, some CoNS species can inhibit pathogenic growth and virulence factors through the secretion of antimicrobial molecules and other mechanisms. CoNSs reside on the skin surface of healthy adults in the range of 10 to 1 ×  10^5^ CFU/cm^3^ [30]. Since the first species of CoNSs were detected by Pasteur and Ogston in 1880, more than 40 species of bacteria, mainly *Staphylococcus epidermidis*, *Staphylococcus hominis*, and *Staphylococcus capitis*, have been identified [31]. These bacteria can secrete antimicrobial peptides (AMPs) that kill *S. aureus* efficiently and precisely. For example, *Staphylococcus epidermidis* secretes sphingomyelinase to break down sphingomyelin into ceramides; *Staphylococcus hominis* secretes micrococcin P1; and *Staphylococcus lugdunensis* secretes the thiazolidin-containing cyclic peptide lugdunin to inhibit the colonization and growth of pathogenic bacteria such as *S. aureus* [8,32]. However, these bacteria may be conditionally pathogenic. *Staphylococcus epidermidis* behaves as an “accomplice” to *S. aureus* when the barrier function is defective or in an inflammatory state [33]. It can damage the barrier function of the skin by releasing the cysteine protease EcpA, which degrades desmoglein-1 and the antimicrobial peptide LL-37 [34]. Currently, most studies on the function of *Staphylococcus* spp. are based on patients with atopic dermatitis (AD). However, the clinical presentation of eczema in elderly individuals is not exactly equivalent to that of AD in old individuals, and only a small proportion of these patients fulfill the diagnostic criteria for AD [35,36]. Therefore, the mechanism of *Staphylococcus* spp. infection in elderly eczema patients should be further analyzed in depth.

In addition to the genus *Staphylococcus*, in this study, we found that other species that have not been frequently reported are worthy of more attention, and their roles in eczema in elderly individuals deserve further investigation. By comparing the differences in skin microbiota between elderly and younger eczema patients, it was found that the two groups had their own skin microbiota composition characteristics. Compared to the young eczema group, the elderly eczema group showed a significant increase in the abundance of five genera among the top 20 most abundant genera (Fig 4 G,H). Among these five genera, *Kaistella* was noteworthy because it was the only genus positively correlated with the EASI score and was among the 20 most abundant genera (Fig 5 A). The genus *Kaistella* is a Gram-stain-negative genus that belongs to the family *Weeksellaceae* and phylum Bacteroidetes [37]. It has been isolated from human clinical and environmental sources, including soil and water [38]. However, researchers have focused mainly on its taxonomy. To the best of our knowledge, the effects of this genus on the skin have never been reported. It has been reported that the genus *Kaistella* can produce esterase that hydrolyze polyethylene terephthalate (PET) plastic [39]. Considering the hydrolytic activity of esterase on lipid [40], we speculated that this genus may interfere with the hydrolysis of lipids on the skin surface through the secretion of esterase, which could explain its significant positive correlation with the EASI score.

Although the abundance of *Streptococcus* did not differ significantly between groups in terms of skin microbiota composition, surprisingly, most species at the genus level were positively correlated according to correlation analyses (Fig 5 B). Streptococci are normally present in healthy skin microbiota and help maintain skin health. However, variations in their abundance may lead to skin diseases. A previous study on the skin microbiota and skin aging showed that the genus *Streptococcus* is susceptible to becoming conditionally pathogenic with age due to the immature and recessive state of the skin barrier in children and elderly individuals, respectively [41]. Combined with the skin characteristics of elderly individuals, we hypothesize that this may be caused by the excessive proliferation of streptococci due to changes in the physiological function of the skin in elderly individuals. Among the genus *Streptococcus*, group A *Streptococcus* (GAS) is the most closely related to humans, and it is widely distributed in mucous membranes and the skin [42]. GAS irreversibly adheres to specific receptors on host cells through adhesins and antigens on filamentous protrusions of the cell wall and reproduces on the skin. The main component of streptococcal adhesin is teichoic acid, and its corresponding receptor is fibronectin on host epithelial cells. Fibronectin is exposed when the skin is broken, and the GAS can subsequently adhere to and multiply on the skin [43]. The aging process of the skin in elderly individuals results in a slower rate of epidermal turnover and a decreased thickness due to the lengthening of the epidermal cell cycle. Aging skin becomes dry, atrophic and loose [44]. These changes make it easier for external irritants and allergens to invade the skin, increasing the probability of skin damage [45]. Moreover, the neurological changes in the skin associated with aging lead to an increase in the nerve thresholds for sensing touch and pain, which can put the skin at increased risk of damage [46]. Increased skin breakdown may eventually lead to an overgrowth of GAS, which produces a variety of virulence factors (e.g., exotoxins, hemolysins, and some enzymes). Similar to the current state of staphylococcal research, although a great deal of research has been done on the taxonomic identification and pathogenic mechanisms of streptococci, there are very few studies based on elderly patients with eczema.

In the above, although *Staphylococcus* has been extensively reported in elderly eczema, other specific genera such as *Kaistella* and *Streptococcus* also deserve attention. Understanding the role of these less-studied bacteria can enhance our therapeutic strategies, as they may contribute to skin barrier dysfunction or inflammatory processes. This awareness encourages clinicians to adopt a more comprehensive approach to treatment, potentially including targeted microbiome modulation or specific antimicrobial therapies [47,48]. Additionally, recognizing the influence of these microorganisms can lead to better patient education about skin care practices and the importance of maintaining a balanced skin microbiome. Ultimately, incorporating these insights into practice may improve outcomes for elderly patients suffering from eczema.

## Conclusion

A decrease in skin structure, immunity and other functional aspects in elderly individuals leads to susceptibility to eczema, which seriously interferes with the quality of life of elderly individuals. However, there is still a lack of sufficient data on the structural characteristics of the skin microbiota of elderly eczema patients, which makes it difficult to provide adequate data for clinical treatment. The rapid development of genomics technology has provided a new opportunity to study the structure and function of the skin microbiota of elderly eczema patients. In this study, the skin microbiota of elderly eczema patients was characterized by amplicon sequencing technology. Many species deserve attention in the future in addition to the frequently reported genus *Staphylococcus*. It is worth noting that this study has certain shortcomings, such as the limited number of patients and the single-center of the study, which may have led to biased results. In the future, the sample size should be increased, and multicenter studies should be carried out to provide a more reliable reference basis for clinical practice.
